# Crystal structure of *catena*-poly[[di­aqua­cadmium(II)]-μ-3,3′-(1,3-phenyl­ene)diacrylato]

**DOI:** 10.1107/S2056989015005411

**Published:** 2015-03-21

**Authors:** Xiao-Ping Zhang, Xin Chen, Kun-Lin Huang

**Affiliations:** a Key Laboratory of Green Synthesis and Applications, College of Chemistry, Chongqing Normal University, Chongqing 401331, People’s Republic of China

**Keywords:** V-shaped ligand, helical chain, chiral coordination polymer, cadmium, crystal structure

## Abstract

In the crystal of the title polymeric complex, [Cd(C_12_H_8_O_4_)(H_2_O)_2_]_*n*_, the Cd^II^ cation, located on a twofold rotation axis, is coordinated by two water mol­ecules and chelated by two phenyl­enediacrylate anions (mpda) in a distorted octa­hedral geometry. The mpda anions bridge the Cd^II^ cations, forming helical chains propagating along the *c-*axis direction. The mpba anion has twofold symmetry with two benzene C atoms located on the twofold rotation axis. In the crystal, O—H⋯O hydrogen bonds link the polymeric helical chains into a three-dimensional supra­molecular architecture.

## Related literature   

For V-shaped metal complexes coordinated by the phenyl­enediacrylate anion, see: Liu *et al.* (2013 ▸). For related metal-organic coordination polymers with an *m*-phenyl­enedi­carboxyl­ate ligand, see: Yang *et al.* (2014 ▸).
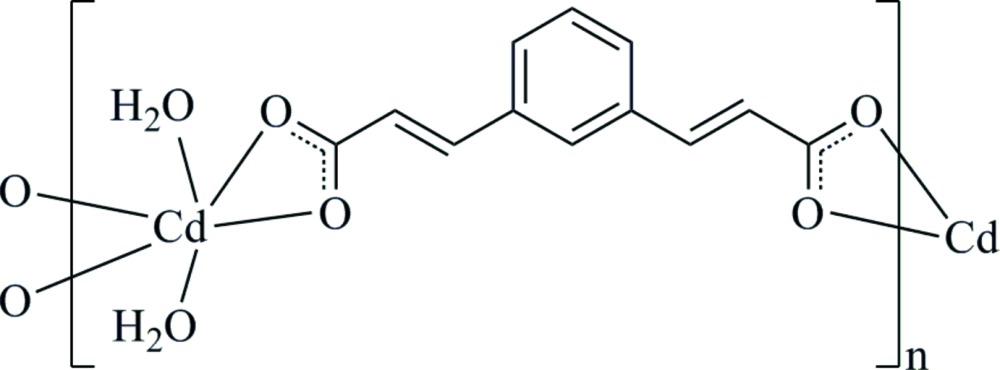


## Experimental   

### Crystal data  

[Cd(C_12_H_8_O_4_)(H_2_O)_2_]
*M* *_r_* = 364.62
Orthorhombic, 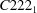

*a* = 5.3145 (12) Å
*b* = 11.991 (3) Å
*c* = 19.767 (5) Å
*V* = 1259.7 (5) Å^3^
*Z* = 4
Mo *K*α radiation
μ = 1.75 mm^−1^
*T* = 293 K
0.35 × 0.32 × 0.25 mm

### Data collection  

Bruker SMART APEXII CCD diffractometer
Absorption correction: multi-scan (*SADABS*; Bruker, 2008 ▸) *T*_min_ = 0.59, *T*_max_ = 0.68
3205 measured reflections
1107 independent reflections
950 reflections with *I* > 2σ(*I*)
*R*_int_ = 0.036

### Refinement  

*R*[*F* ^2^ > 2σ(*F* ^2^)] = 0.039
*wR*(*F* ^2^) = 0.155
*S* = 1.08
1107 reflections
96 parameters
2 restraints
H atoms treated by a mixture of independent and constrained refinement
Δρ_max_ = 0.93 e Å^−3^
Δρ_min_ = −0.64 e Å^−3^
Absolute structure: Flack (1983 ▸), 1035 Friedel pairs
Absolute structure parameter: 0.07 (19)

### 

Data collection: *APEX2* (Bruker, 2008 ▸); cell refinement: *SAINT* (Bruker, 2008 ▸); data reduction: *SAINT*; program(s) used to solve structure: *SHELXS97* (Sheldrick, 2008 ▸); program(s) used to refine structure: *SHELXL97* (Sheldrick, 2008 ▸); molecular graphics: *DIAMOND* (Brandenburg, 2008 ▸)); software used to prepare material for publication: *PLATON* (Spek, 2009 ▸).

## Supplementary Material

Crystal structure: contains datablock(s) I, global. DOI: 10.1107/S2056989015005411/xu5838sup1.cif

Structure factors: contains datablock(s) I. DOI: 10.1107/S2056989015005411/xu5838Isup2.hkl

**Fig. 1.**
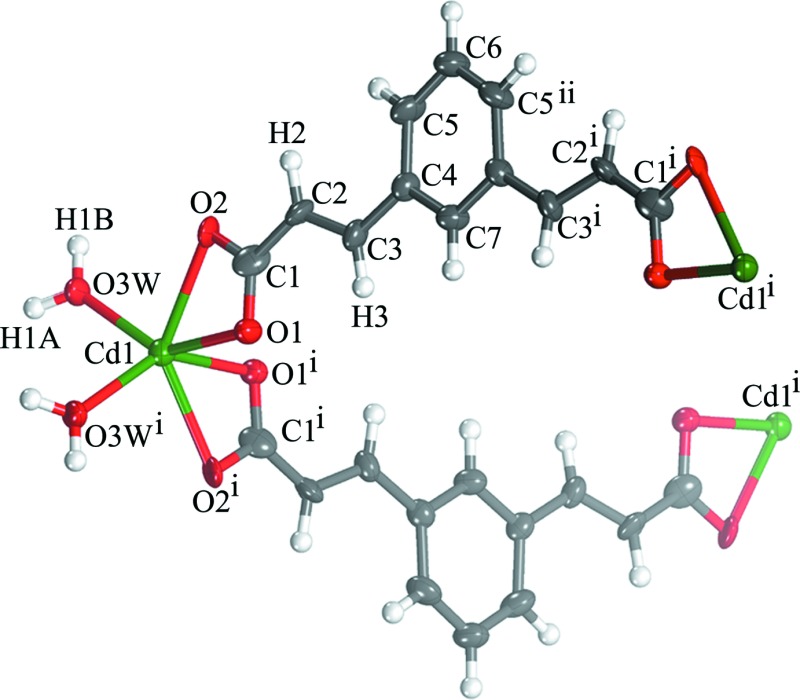
Part of the crystal structure of the mpda ligand and CdII centres in (1), showing the atom-numbering scheme. Displacement ellipsoids are drawn at the 30% probability level. [Symmetry codes: (i) x, -y + 2, -z + 2; (ii) -x + 2, y, -z + 3/2].

Click here for additional data file.II 1 x y z x y z . DOI: 10.1107/S2056989015005411/xu5838fig1.tif
Part of the crystal structure of the mpda ligand and Cd^II^ centres in (**1**), showing the atom-numbering scheme. Displacement ellipsoids are drawn at the 30% probability level. [Symmetry codes: (i) *x*, −*y* + 2, −*z* + 2; (ii) −*x* + 2, *y*, −*z* + 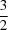].

CCDC reference: 1054223

Additional supporting information:  crystallographic information; 3D view; checkCIF report

## Figures and Tables

**Table 1:** Selected bond lengths ()

Cd1O1	2.329(6)
Cd1O2	2.380(6)
Cd1O3*W*	2.199(7)

**Table 2:** Hydrogen-bond geometry (, )

*D*H*A*	*D*H	H*A*	*D* *A*	*D*H*A*
O3*W*H1*A*O1^i^	0.85(2)	1.88(4)	2.695(9)	160(8)
O3*W*H1*B*O2^ii^	0.84(2)	1.92(3)	2.736(8)	162(8)

Symmetry codes: (i) 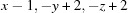; (ii) 
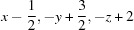
.

## supplementary crystallographic information

### S1. Introduction

In crystal engineering, a great inter­est is focused on the supra­molecular self-assembly of helical or chiral coordination polymers from appropriately bridging ligands and metallic tectons through either coordinate bonds and hydrogen bonds. Although much effort has been devoted to the assembly of helical or chiral coordination polymers based on chiral or achiral ligands, the design and construction of chiral coordination polymer based on helical topology is still a great challenge for chemists.

### S2. Experimental

All reagents and solvents employed were commercially available and were used as received without further purification.

### S2.1. Synthesis and crystallization

A mixture of Cd(NO_3_)_2_·4H_2_O (62 mg, 0.25 mmol), m-phenyl­enediacrylic acid (H_2_mpda, 44 mg, 0.2 mmol) and H_2_O (10 mL) was sealed in a 25 mL stainless steel reactor with a Teflon liner, heated to 413 K for 3 d and then cooled to room temperature. The crystals were washed with methanol to give the title complex in about 35% yield (based on the H_2_mpda ligand).

### S2.2. Refinement

All non-hydrogen atoms are easily found from the different Fourier maps and refined anisotropically. All H atoms of H_2_O molecules are found in the difference Fourier map. The C-bound H atoms of aromatic rings were refined using a riding model [0.93 Å (CH) and U_iso_(H) = 1.2U_eq_(C)].

### S3. Results and discussion

The m-phenyl­enediacrylate (mpda) has various conformations for the rotation of carbon-carbon bonds, such as the C—C single bonds between aromatic rings and C=C bonds. The V-shaped mpda coordinated with metal ions have been documented (Liu *et al.*, 2013). But the mpda has not been well exploited in constructing metal-organic coordination polymers in comparison with the m-phenyl­enedi­carboxyl­ate ligand (Yang *et al.*, 2014). Herein, we report a poly[(m-phenyl­enediacrylate)(water)cadmium] with the formulae [Cd(mpda)(H_2_O)_2_]*_n_* (1). The title compound crystallizes in the chiral *C*222 (1) space group, and contains a homochiral left-handed single-stranded helical chain.

In the structure of 1 (Fig. 1), the Cd^2+^ centre adopts a six-coordinated mode with a distorted o­cta­hedral geometry (CdO_6_) *via* binding to two water O atoms and four O atoms from two different CO_2_^-^ groups of mpda ligands [O1, O2, O1^i^, O2^i^; symmetry code: (i) x,-y+2,-z+2]. The Cd—O distances range from 2.199 (7) to 2.380 (6) Å. Taking account of the O3W—Cd1—O3W^i^ angle of 92.1 (4)°, the coordination mode of Cd^2+^ ion may play an determining role in the formation of the Cd1^i^···Cd1···Cd1^i^ angle of 85.65°. Each mpda ligand with a monodentate mode bridges two different Cd^2+^ ions, via carboxyl­ate groups at each end of the ligand molecule. Of the structure of the mpda, two acrylate groups (CH=CH-COO^-^) are located at the benzene ring in a *trans*-position fashion. The dihedral angles of the CH=CH-COO^-^ and C_6_H_4_—CH=CH group are *ca.*11.4° and 7.9°, respectively, while that of C1···C7···C6···C1^i^ is 180°. These dihedral angle data indicate that the mpda ligand is nearly coplanar and there exist intra­molecular π-π electronic conjugations.

The mpda ligands serve as V-shaped bridges to link the metal ions into infinite helical coordination chains running along the *c*-axis, with the Cd···Cd distance separated by mpda being 14.54 Å. The helical pitch of 19.767 (5) Å is equal to the *c* dimension of the unit cell. There has the one and only left-handed single-stranded chain in 1, which reveals that this work presents a new unusual example of homochiral architecture based on the helical topology. In 1, the packing of the helical chains is sustained by complicated hydrogen bonds and π–π stacking involving all the coordinated water molecules, and the C=C double bonds and phenyl­ene rings of the mpda ligands. Each phenyl­ene ring from one chain inter­acts with a C=C double bond from another chain, with the inter­plane distances being 3.53 Å. Careful examination of the structure indicates that strong inter­chain hydrogen bonds of O3W—H1A···O1^iii^ [2.695 (6) Å; Symmetry code: (iii) x-1, -y+2, -z+2] exist between the two independent chains. It is clear that the hydrogen (H1A) atoms of one coordinated water molecule serve as the acceptors, while the oxygen (O1^iii^) atoms of the mpda ligands serve as the donors in each self-recognition site. The above-mentioned π–π and O3W—H1A···O1^iii^ inter­actions bring the helical coordination chains into wave-shaped two-dimensional layers. Further a three-dimensional supra­molecular architecture is result from wave-shaped motifs through the inter­layer hydrogen bonds of O3W—H1B···O2^iv^ [2.736 (8) Å; Symmetry code: (iv) x-1/2, -y+3/2, -z+2].

### Crystal data

**Table:**

[Cd(C_12_H_8_O_4_)(H_2_O)_2_]	*F*(000) = 720
*M**_r_* = 364.62	*D*_x_ = 1.922 Mg m^−^^3^
Orthorhombic, *C*222_1_	Mo *K*α radiation, λ = 0.71073 Å
Hall symbol: C 2c 2	Cell parameters from 1107 reflections
*a* = 5.3145 (12) Å	θ = 2.0–25.0°
*b* = 11.991 (3) Å	µ = 1.75 mm^−^^1^
*c* = 19.767 (5) Å	*T* = 293 K
*V* = 1259.7 (5) Å^3^	Block, colorless
*Z* = 4	0.35 × 0.32 × 0.25 mm

### Data collection

**Table:**

Bruker SMART APEXII CCD diffractometer	1107 independent reflections
Radiation source: fine-focus sealed tube	950 reflections with *I* > 2σ(*I*)
Graphite monochromator	*R*_int_ = 0.036
ω scans	θ_max_ = 25.0°, θ_min_ = 2.1°
Absorption correction: multi-scan (*SADABS*; Bruker, 2008)	*h* = −5→6
*T*_min_ = 0.59, *T*_max_ = 0.68	*k* = −13→14
3205 measured reflections	*l* = −19→23

### Refinement

**Table:**

Refinement on *F*^2^	Secondary atom site location: difference Fourier map
Least-squares matrix: full	Hydrogen site location: inferred from neighbouring sites
*R*[*F*^2^ > 2σ(*F*^2^)] = 0.039	H atoms treated by a mixture of independent and constrained refinement
*wR*(*F*^2^) = 0.155	*w* = 1/[σ^2^(*F*_o_^2^) + (0.1*P*)^2^ + 7.5572*P*] where *P* = (*F*_o_^2^ + 2*F*_c_^2^)/3
*S* = 1.08	(Δ/σ)_max_ < 0.001
1107 reflections	Δρ_max_ = 0.93 e Å^−^^3^
96 parameters	Δρ_min_ = −0.64 e Å^−^^3^
2 restraints	Absolute structure: Flack (1983), 1035 Friedel pairs
Primary atom site location: structure-invariant direct methods	Absolute structure parameter: 0.07 (19)

### Special details

**Table:**

Geometry. All e.s.d.'s (except the e.s.d. in the dihedral angle between two l.s. planes) are estimated using the full covariance matrix. The cell e.s.d.'s are taken into account individually in the estimation of e.s.d.'s in distances, angles and torsion angles; correlations between e.s.d.'s in cell parameters are only used when they are defined by crystal symmetry. An approximate (isotropic) treatment of cell e.s.d.'s is used for estimating e.s.d.'s involving l.s. planes.
Refinement. Refinement of *F*^2^ against ALL reflections. The weighted *R*-factor *wR* and goodness of fit *S* are based on *F*^2^, conventional *R*-factors *R* are based on *F*, with *F* set to zero for negative *F*^2^. The threshold expression of *F*^2^ > σ(*F*^2^) is used only for calculating *R*-factors(gt) *etc*. and is not relevant to the choice of reflections for refinement. *R*-factors based on *F*^2^ are statistically about twice as large as those based on *F*, and *R*- factors based on ALL data will be even larger.

### Fractional atomic coordinates and isotropic or equivalent isotropic displacement parameters (Å^2^)

**Table:**

	*x*	*y*	*z*	*U*_iso_*/*U*_eq_	
Cd1	−0.00336 (16)	1.0000	1.0000	0.0311 (3)	
O1	0.2947 (11)	0.9898 (5)	0.9139 (3)	0.0332 (13)	
O2	0.1598 (11)	0.8310 (5)	0.9546 (3)	0.0335 (14)	
O3W	−0.2906 (13)	0.8960 (5)	1.0493 (4)	0.0438 (18)	
C1	0.307 (2)	0.8841 (8)	0.9156 (4)	0.040 (2)	
C2	0.4734 (19)	0.8224 (7)	0.8726 (5)	0.039 (2)	
H2	0.4462	0.7460	0.8689	0.046*	
C3	0.6643 (18)	0.8655 (8)	0.8375 (4)	0.0329 (19)	
H3	0.6894	0.9420	0.8411	0.040*	
C4	0.8387 (19)	0.8040 (7)	0.7936 (4)	0.0297 (19)	
C5	0.848 (2)	0.6888 (8)	0.7930 (5)	0.041 (2)	
H5	0.7472	0.6494	0.8231	0.049*	
C6	1.0000	0.6307 (10)	0.7500	0.042 (3)	
H6	1.0000	0.5531	0.7500	0.050*	
C7	1.0000	0.8616 (10)	0.7500	0.033 (3)	
H7	1.0000	0.9392	0.7500	0.039*	
H1A	−0.436 (7)	0.917 (7)	1.062 (4)	0.02 (2)*	
H1B	−0.288 (15)	0.826 (2)	1.056 (4)	0.01 (2)*	

### Atomic displacement parameters (Å^2^)

**Table:**

	*U*^11^	*U*^22^	*U*^33^	*U*^12^	*U*^13^	*U*^23^
Cd1	0.0265 (5)	0.0292 (5)	0.0377 (5)	0.000	0.000	−0.0032 (3)
O1	0.029 (3)	0.024 (3)	0.046 (3)	−0.001 (3)	0.007 (3)	−0.001 (3)
O2	0.012 (3)	0.027 (3)	0.062 (4)	−0.006 (3)	0.013 (3)	−0.004 (3)
O3W	0.024 (4)	0.028 (3)	0.079 (5)	−0.002 (3)	0.015 (4)	0.001 (4)
C1	0.053 (6)	0.040 (5)	0.027 (4)	−0.009 (5)	0.000 (5)	−0.009 (4)
C2	0.031 (5)	0.026 (4)	0.059 (6)	−0.015 (5)	0.006 (5)	−0.007 (4)
C3	0.024 (4)	0.037 (5)	0.038 (5)	−0.008 (4)	0.005 (4)	−0.001 (4)
C4	0.030 (4)	0.031 (5)	0.028 (4)	−0.004 (5)	0.004 (4)	0.002 (3)
C5	0.046 (6)	0.030 (5)	0.046 (5)	−0.012 (5)	0.008 (5)	0.004 (4)
C6	0.048 (8)	0.024 (6)	0.054 (7)	0.000	0.008 (8)	0.000
C7	0.041 (7)	0.025 (6)	0.032 (6)	0.000	0.006 (7)	0.000

### Geometric parameters (Å, º)

**Table:**

Cd1—O1^i^	2.329 (6)	C2—C3	1.334 (13)
Cd1—O1	2.329 (6)	C2—H2	0.9300
Cd1—O2^i^	2.380 (6)	C3—C4	1.468 (12)
Cd1—O2	2.380 (6)	C3—H3	0.9300
Cd1—O3W	2.199 (7)	C4—C5	1.383 (14)
Cd1—O3W^i^	2.199 (7)	C4—C7	1.398 (10)
Cd1—C1^i^	2.727 (10)	C5—C6	1.363 (12)
O1—C1	1.270 (12)	C5—H5	0.9300
O2—C1	1.269 (12)	C6—C5^ii^	1.363 (12)
O3W—H1A	0.85 (2)	C6—H6	0.9300
O3W—H1B	0.84 (2)	C7—C4^ii^	1.398 (10)
C1—C2	1.432 (14)	C7—H7	0.9300
			
O3W—Cd1—O3W^i^	92.1 (4)	Cd1—O3W—H1B	128 (6)
O3W—Cd1—O1^i^	100.3 (3)	H1A—O3W—H1B	105 (9)
O3W^i^—Cd1—O1^i^	140.0 (2)	O2—C1—O1	119.0 (9)
O3W—Cd1—O1	140.0 (2)	O2—C1—C2	118.8 (9)
O3W^i^—Cd1—O1	100.3 (3)	O1—C1—C2	122.1 (9)
O1^i^—Cd1—O1	94.3 (3)	C3—C2—C1	125.3 (9)
O3W—Cd1—O2^i^	124.6 (3)	C3—C2—H2	117.3
O3W^i^—Cd1—O2^i^	86.4 (2)	C1—C2—H2	117.3
O1^i^—Cd1—O2^i^	55.3 (2)	C2—C3—C4	126.4 (8)
O1—Cd1—O2^i^	94.2 (2)	C2—C3—H3	116.8
O3W—Cd1—O2	86.4 (2)	C4—C3—H3	116.8
O3W^i^—Cd1—O2	124.6 (3)	C5—C4—C7	117.8 (9)
O1^i^—Cd1—O2	94.2 (2)	C5—C4—C3	122.0 (9)
O1—Cd1—O2	55.3 (2)	C7—C4—C3	120.2 (8)
O2^i^—Cd1—O2	137.3 (3)	C6—C5—C4	122.6 (10)
O3W—Cd1—C1^i^	116.0 (3)	C6—C5—H5	118.7
O3W^i^—Cd1—C1^i^	113.7 (3)	C4—C5—H5	118.7
O1^i^—Cd1—C1^i^	27.7 (3)	C5—C6—C5^ii^	118.5 (12)
O1—Cd1—C1^i^	93.6 (3)	C5—C6—H6	120.7
O2^i^—Cd1—C1^i^	27.7 (3)	C5^ii^—C6—H6	120.7
O2—Cd1—C1^i^	116.4 (3)	C4^ii^—C7—C4	120.8 (11)
C1—O1—Cd1	93.9 (6)	C4^ii^—C7—H7	119.6
C1—O2—Cd1	91.5 (5)	C4—C7—H7	119.6
Cd1—O3W—H1A	127 (6)		

Symmetry codes: (i) *x*, −*y*+2, −*z*+2; (ii) −*x*+2, *y*, −*z*+3/2.

### Hydrogen-bond geometry (Å, º)

**Table:**

*D*—H···*A*	*D*—H	H···*A*	*D*···*A*	*D*—H···*A*
O3*W*—H1*A*···O1^iii^	0.85 (2)	1.88 (4)	2.695 (9)	160 (8)
O3*W*—H1*B*···O2^iv^	0.84 (2)	1.92 (3)	2.736 (8)	162 (8)

Symmetry codes: (iii) *x*−1, −*y*+2, −*z*+2; (iv) *x*−1/2, −*y*+3/2, −*z*+2.
