# Supplementary figures and images for: Crystal structure of catena-poly[[di­aqua­cadmium(II)]-μ-3,3′-(1,3-phenyl­ene)diacrylato]
Source: Acta Crystallogr E Crystallogr Commun. 2015 Mar 21;71(Pt 4):m91–2. doi: 10.1107/S2056989015005411 (PMC4438799; doi:10.1107/S2056989015005411)

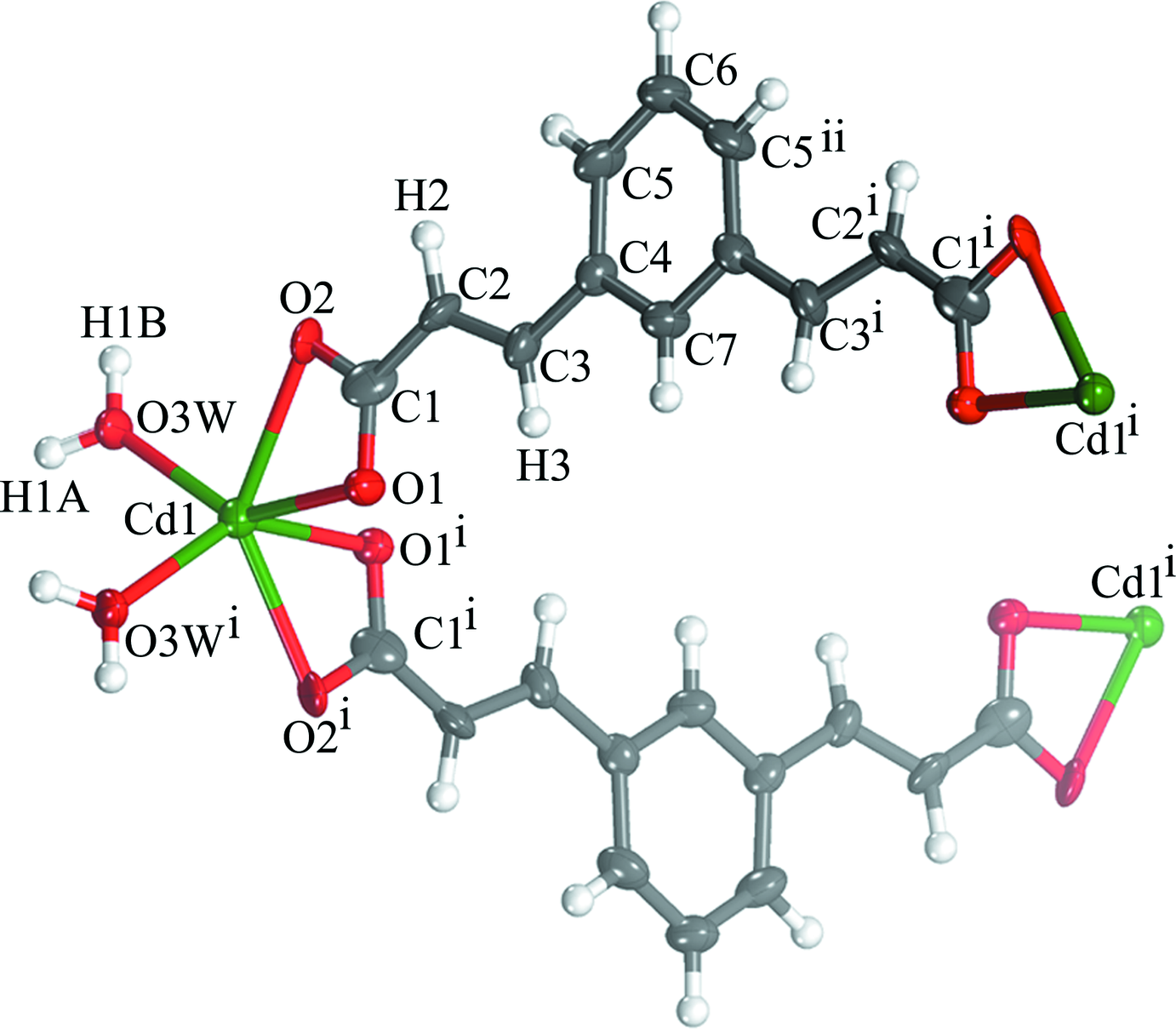

Supplement: Supplementary file 3 [file e-71-00m91-fig1.tif]
